# Decreased biventricular myocardial deformation in fetuses with lower urinary tract obstruction

**DOI:** 10.1186/s12884-020-03152-y

**Published:** 2020-08-12

**Authors:** Ran Xu, Jiawei Zhou, Qichang Zhou, Shi Zeng

**Affiliations:** 1 Department of Urology, The Second Xiangya Hospital, Central South University, 410011 Changsha, Hunan China; 2 Department of Ultrasound Diagnosis, The Second Xiangya Hospital, Central South University, 139 Renmin Road (M), Hunan 410011 Changsha, China

**Keywords:** lower urinary tract obstruction, myocardial deformation, strain, strain rate, fetus

## Abstract

**Background:**

To observe myocardial deformations in fetuses with isolated lower urinary tract obstruction (LUTO) and identify the correlation between myocardial deformation and the severity of obstruction.

**Methods:**

The strain (S), strain rate in systole (SRs) and strain rate in diastole (SRd) of the left and right ventricles at the first examination were prospectively analyzed and compared between fetuses with isolated LUTO and gestational age (GA)-matched normal control fetuses. Multiple regression analyses were used to assess the obstructive factors for impaired strain and strain rate, and the independent variables included bladder volume, sum of the bilateral pelvic diameters, sum of the bilateral ureteral diameters, mean bilateral renal artery pulsatility index, and amniotic fluid index.

**Results:**

Thirty-six fetuses with isolated LUTO and 36 normal controls were enrolled. Overall, decreased S, SRs and SRd of both ventricles were noted in fetuses with LUTO (*p* < 0.001). Moreover, S and SR were significantly negatively related to distended bladder volume (*p* < 0.001).

**Conclusions:**

Fetuses with LUTO demonstrated decreased left and right myocardial deformation, and this impaired cardiac dysfunction was correlated with the urinary bladder volume. Evaluating the myocardial deformation in fetal LUTO could provide information to aid in parental counselling and intervention monitoring.

## Background

Lower urinary tract obstruction (LUTO), which occurs in between 2.2 and 3.3 per 10,000 births [1], refers to a heterogeneous group of urinary anatomic malformations with primary lesions on the bladder neck or urethra. Although the diagnostic accuracy is challenging, fetal LUTO can be detected by screening ultrasonography during the late first and second trimesters of pregnancy, characteristically presenting megacystis, various degrees of hydronephrosis and dilated ureters, macro- or microcysts in the kidney parenchyma and oligohydramnios [2, 3]. Untreated fetal LUTO has up to a 45% perinatal mortality rate and approximately 30% morbidity in survivors [4, 5], principally due to lung hypoplasia and renal failure.

Cardiovascular dysfunction in the LUTO, triggered by an increased afterload accompanied by bladder compression and a compressed iliac artery, involves reduced pulmonary venous return due to lung dysplasia and the stimulation of neuro-hormonal mechanisms and may contribute to renal dysfunction and worse outcomes. However, few reports have focused on the cardiovascular effects in fetuses with LUTO. Cardiomegaly, pericardial effusion and ventricular hypertrophy are common in fetal LUTO [6–8]. Right ventricular filling compliance, such as a decreased tricuspid valve E/A ratio [6] and longer right ventricular isovolumetric relaxation time [8], as well as a worse left ventricular myocardial performance index [7], have been noted. To date, there are no detailed data on the characteristics of myocardial deformations in fetuses with isolated LUTO. Therefore, in this study, we aimed to (1) observe biventricular myocardial deformations in fetuses with isolated LUTO with giant bladder and (2) identify the correlation between myocardial deformation and the severity of LUTO.

## Methods

**Fig. 1:**
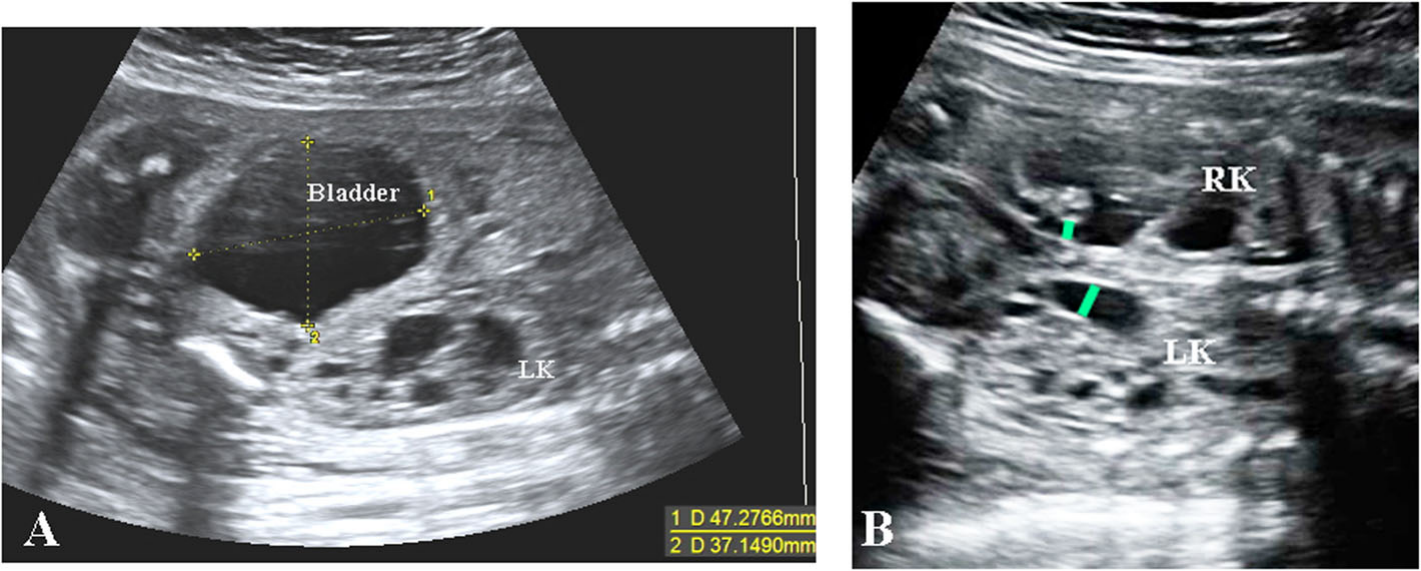
Ultrasonic features of fetal LUTO showed a distended fetal urinary bladder (**a**), fetal hydronephrosis and ureterectasis (**b**, green lines identify bilateral dilated ureters). RK, right kidney; LK, left kidney

A prospective cross-section observational study was conducted at the Second XiangYa Hospital of Central South University in China from January 2015 to December 2018. The study population consisted of pregnant women with an ultrasonic appearance of fetal LUTO, including a distended fetal urinary bladder (enlarged bladder failing to empty over a period of 45 min from the second trimester onward [9]), fetal hydronephrosis and ureterectasis (Fig. 1). We excluded suspected LUTO fetuses of less than 18 gestation weeks due to the hard-acquiring heart image. The other exclusion criteria were as follows: presence of abnormalities in other organ systems; chromosomal abnormalities; and associated maternal complications, such as gestational diabetes, preeclampsia, and thyroid disease. Only the initial examination data were used to analyze if more than one scan was performed on the fetuses. None of the fetuses had any intervention done prior to initial evaluation (i.e. vesicocentesis). The perinatal outcome was noted. The etiology of the LUTO was determined based on the autopsy or postanatal examinations, such as physical check-up, urethroscope, voiding cystourethrography or surgical pathology. The normal control group was randomly collected from nonconsecutive, low-risk, gestational age (GA)-matched healthy pregnant women. Written informed consent was obtained from all families, and this study was approved by the institutional review board at the Second Xiangya Hospital of Central South University.

Screening obstetrical ultrasound was performed for all fetuses by one investigator (Z.S.) using a Sequoia 512 ultrasound system with a 6C2 transducer at 2.5–6.0 MHz (Siemens Medical Solutions USA, Inc., Mountain View, CA, USA). The three diameters of the urinary bladder were measured and used to calculate the bladder volume using the following formula: longitudinal diameter × transverse diameter × anteroposterior diameter × π/6 [10]. The pulsatility index (PI) of the renal arteries was measured in the renal arterial trunk away from the aorta and before any visible branches on a coronal view of the fetal abdomen, with an angle of insonation as close as possible to 0°. The presence of renal cortical cysts, the anteroposterior diameter of the pelvis, ureteral diameter, amniotic fluid index (AFI), PI of the middle cerebral artery (MCA) and umbilical artery (UI) were also recorded.

Fetal echocardiograms were performed at the same time after obstetrical ultrasound screening for all fetuses by one investigator (Z.QC) who was blinded to the group status. Standard and multiple views of the fetal heart were obtained [11], and specific attention was paid to the following issues for the purpose of this study: (1) cardiac/thoracic ratio (C/T ratio), presence of pericardial effusion (defined as fluid ≥ 2 mm during diastole), thickness of the left and right ventricle; (2) presence of fusion of the E wave with the A wave across both mitral and tricuspid valves; and (3) presence of holosystolic mitral and tricuspid regurgitation. The myocardial deformation was performed by one observer who was blinded to the group status and echocardiography manifestation using vector velocity imaging software (VVI; Siemens Medical Solutions, Olympia, Washington). Briefly, as our previously reported [12, 13], a high-quality cine (40–50 frames/sec) of the apical four-chamber view was digitally stored, followed by identification of the cardiac cycle using M-mode and tracing the ventricular endocardium. The myocardial deformation parameters, including the strain (S) and strain rate for systole (SRs) and the strain rate for diastole (SRd), were automatically calculated. S, SRs and SRd of both the left ventricle (LV) and right ventricle (RV) were recorded in this investigation.

Data are presented as the mean ± SD or frequencies (percentage), as appropriate. Data were compared between normal controls and fetuses with LUTO using Student’s t-tests or Fisher’s exact test. After testing the correlations between myocardial deformation parameters and the severity of obstruction in the fetuses with LUTO using Pearson’s correlation coefficient, multiple regression analyses with the stepwise method were calculated to identify the factors for impaired strain and strain rate; the independent variables included mean bilateral renal artery PI, bladder volume, sum of the bilateral pelvic diameters, sum of the bilateral ureteral diameters and AFI. Two-sided P < 0.05 was considered significant. All statistical analyses were performed using PASW statistics software [PASW (SPSS) statistics 18.0, IBM].

## Results

A total of 59 fetuses with suspected LUTO were initially enrolled, but 23 cases were excluded, including 2 with excessive fetal movement resulting in poor echocardiographic images, 11 without follow-up, 6 with postnatally confirmed nonobstructive lesions (four infants with vesicoureteral reflux and two with normal micturition) and 4 with congenital syndrome (two infants with megacystis-microcolon-intestinal hypoperistalsis syndrome, one with macrosomia syndrome and one with prune belly syndrome). Finally, 36 fetuses with isolated LUTO and 36 GA-matched normal controls were studied. The GA at the initial examination was 21.5 ± 1.2 weeks. The perinatal outcomes of fetuses with LUTO are summarized in Table 1.

**Table 1:** The outcome of fetuses with LUTO (*n* = 36)

	Fetus with LUTO (*n* = 36)
Postnatal diagnosis:	
PUV, n (%)	20 (55.5%)
AUV, n (%)	5 (13.9%)
UA, n (%)	5 (13.9%)
US, n (%)	4 (11.1%)
MS, n (%)	2 (5.5%)
Outcome of pregnancy	
TOP, n (%)	14 (38.8%)
IUFD, n (%)	3 (8.3%)
Live birth, n (%)	19 (52.8%)
Postnatal treatment and outcome	
Follow-up period, month	30.9 ± 19.6
Endoscopic surgery, n (%)	19 (100%)
^ a^Renal dysfunction, n (%)	3 (15.8%)

^a^was defined as KDOQI CKD2 or higher (eGFR < 90 ml/min/1.73 m^2^) at the end of follow up

*PUV* posterior urethral valves; *AUV *anterior urethral valves; *UA *urethral atresia; *US* urethral stenosis; *MS* meatal stenosis; *TOP* termination of pregnancy; *IUFD* intrauterine fetal death

Compared with controls, the fetuses with LUTO undoubtedly had increased bladder volume, dilated pelvic and ureteral diameters and reduced amniotic fluid volume (*p* < 0.001). Eighteen (50%) fetuses with LUTO showed classic keyhole signs. Although 8 fetuses with LUTO (22.2%) demonstrated cortical renal cysts, the mean PI of the bilateral renal artery did not differ.

**Fig. 2:**
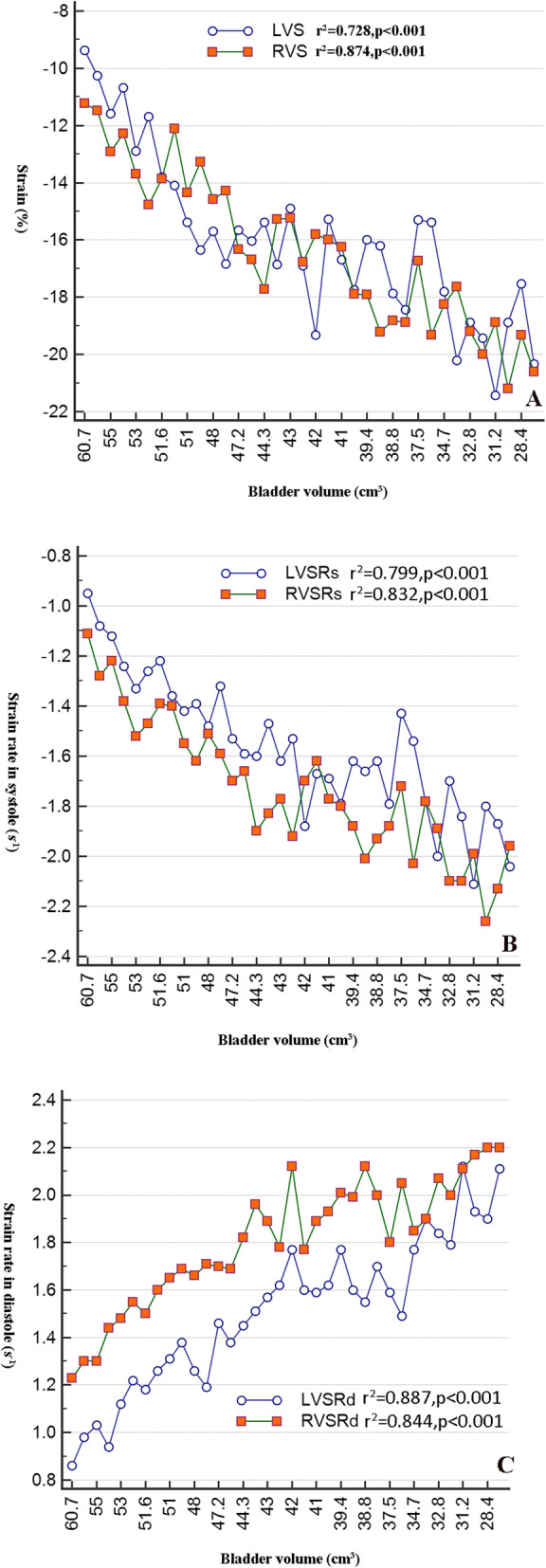
The scatter plot shows that a distended bladder volume was significantly related to decreased strain (**a**), strain rate in systole (**b**) and strain rate in diastole (**c**) of both ventricles in fetuses with LUTO. S, strain; SRs, strain rate in systole; SRd, strain rate in diastole; LV, left ventricle; RV, right ventricle

Regarding echocardiogram manifestations, fetuses with LUTO demonstrated an increased C/T ratio (*p* < 0.001), more cases of RV hypertrophy and blood flow fusion across the tricuspid valve (*p* < 0.05). Moreover, the fetuses with LUTO demonstrated overall decreased myocardial deformation in both the left and right ventricles, including the strain and strain rate in systole and diastole (*p* < 0.001, Table 2). Multiple regression analyses showed that the strain and strain rate of both ventricles were significantly negatively related to distended bladder volume (*p* < 0.001, Fig. 2).

**Table 2:** The ultrasonic manifestation and myocardial deformation in the cohort of studies (*n* = 72)

	Fetus with LUTO (*n* = 36)	Normal control (*n* = 36)	P
Maternal age, years	30 ± 6	29 ± 4	0.36
GA at initial examination, weeks	21.5 ± 1.2	21.5 ± 1.2	1
EFW at initial examination, g	404.5 ± 89.3	416.2 ± 82.3	0.92
Male, n (%)	34 (94%)	25 (69.4%)	0.01
Obstruction manifestation			
Bladder volume, cm^3^	42.7 ± 8.6	8.6 ± 3.8	< 0.001
Keyhole sign, n (%)	18 (50%)	0	< 0.001
Pelvic diameters^a^, mm	18 ± 4	2 ± 2	< 0.001
Ureteral diameters^b^, mm	4 ± 1	0	< 0.001
RA-PI^c^	2.00 ± 0.10	1.97 ± 0.14	0.28
Renal cortical cysts, n (%)	8 (22.2%)	0	< 0.01
Oligo-/anhydramnios, n (%)	22 (61.1%)	0	< 0.001
AFI	42 ± 36	138 ± 26	< 0.001
Echocardiogram			
C/T	0.35 ± 0.06	0.30 ± 0.02	< 0.001
PE, n (%)	5 (13.9%)	0	0.05
LV hypertrophy, n (%)	4 (11.1%)	0	0.11
LV thickness, mm	2.0 ± 0.4	1.9 ± 0.3	0.09
RV hypertrophy, n (%)	6 (16.7%)	0	0.03
RV thickness, mm	2.1 ± 0.4	1.9 ± 0.2	0.05
MVR, n (%)	3 (8.3%)	0	0.24
TVR, n (%)	5 (13.9%)	0	0.05
MV fusion, n (%)	4 (11.1%)	0	0.11
TV fusion, n (%)	7 (19.4%)	0	0.01
Myocardial deformation			
LV-S, %	-16.03 ± 2.89	-20.21 ± 1.61	< 0.001
LV-SRs, s^− 1^	-1.57 ± 0.27	-2.07 ± 0.17	< 0.001
LV-SRd, s^− 1^	1.51 ± 0.32	1.95 ± 0.43	< 0.001
RV-S, %	-16.35 ± 2.70	-26.42 ± 1.96	< 0.001
RV-SRs, s^− 1^	-1.73 ± 0.27	-2.61 ± 0.81	< 0.001
RV-SRd, s^− 1^	1.81 ± 0.26	2.54 ± 0.56	< 0.001

^a^The sum of the anteroposterior diameter of the bilateral pelvis; ^b^sum of the bilateral ureteral diameters;^c^The mean of the bilateral renal artery pulsatility index;

*GA* gestational age; *EFW* estimated fetal weight; *RA* renal artery; *AFI* amniotic fluid index; *C/T* cardiothoracic ratio; *PE* pericardial effusion; *LV* left ventricle; *RV* right ventricle; *MVR* mitral valve regurgitation; *TVR* tricuspid valve regurgitation; *S* strain; *SRs* strain rate in systole; *SRd* strain rate in diastole

## Discussion

The effect on the myocardium in the LUTO group was present in utero, demonstrated as an overall decreased strain and strain rate in both the left and right ventricles, as well as negative correlations with the degree of obstruction.

Cardiac geometry in fetuses with LUTO showed an increased C/T ratio and ventricular hypertrophy, similar to previous reports [6, 7]. RV diastolic dysfunction in fetal LUTO was previously observed, reflected as a decreased tricuspid valve E/A ratio [6] and a longer RV isovolumetric relaxation time [8]. LV diastolic dysfunction was previously demonstrated as a shorter mitral valve (MV) inflow time and higher LV MPI [7]. Our study showed significantly decreased strain and strain rates in systole and diastole of both ventricles, indicating overall myocardial dysfunction in fetal LUTO.

Hemodynamic alterations in fetal LUTO contributes to cardiac dysfunction. On the one hand, both the afterload and preload of the RV were found to be increased in the setting of LUTO. The extended bladder could mechanically compress the iliac arteries and the origins of the umbilical arteries and cause an increased afterload of the RV. Such vascular compression and increased impedance in the fetus with LUTO have been observed by Rychik J [6], showing a lower PI in the lower distal descending aorta and bilateral iliac arteries. Increased preload is caused by renin–angiotensin–aldosterone system (RAAS)‑mediated fluid overload. Experiments on fetal sheep [14] demonstrated that fetal partial ureteral obstruction can cause upregulation of the levels of renin, angiotensinogen and angiotensin receptors, which result in water and salt retention. On the other hand, the preload of the LV was decreased due to reduced pulmonary vein return associated with lung hypolasia, which is common in fetal LUTO. Such a diminished preload of the LV in fetal LUTO has been observed by Cohen J [7], showing a reduced LV cardiac index, and could result in diminished forward flow to the coronary arteries and less myocardial perfusion.

Other contributions to the overall cardiac dysfunction are triggered by obstructive uropathy. Renal damage and loss of renal function have been observed in fetuses with LUTO through fetal histopathology and urinary/serum tests. Fetal and neonatal animal models of ureteral obstruction have demonstrated decreased renal mass, reduced number of nephrons, loss of tubular cells and collecting ducts, a decreased glomerular number and glomerular filtration rate, and increased interstitial fibrosis [15, 16]. Furthermore, human fetal autopsy has revealed higher rates of mesenchymal and tubular apoptosis [17]. Abnormal fetal urinary/serum examinations in LUTO, reflected as increased urinary β-2-microglobulin [18], urinary sodium and calcium [18], and 12 fetal urinary peptides [19], as well as serum β-2-microglobulin [20], have been observed and noted to have strong predictive value for poor postnatal renal function. The renal dysfunction in fetal LUTO, through a number of pathways [21] relevant to hormonal responses, inflammation/oxidative stress and metabolic changes, can induce cardiac fibroblast proliferation[22], endothelial dysfunction [23] and cardiac remodeling[24, 25]and eventually cause overall cardiac dysfunction.

Our study found that fetuses with severe bladder extension were associated with worse myocardial strain and strain rates. On the one hand, the larger the urinary bladder was, the higher the compression on the iliac artery and the intraabdominal pressure were. As a result, the hemodynamic contributor to cardiac malfunction was magnified. On the other hand, the larger the urine bladder was, the worse was the renal dysfunction presented. Recently, Fontanella F [26] found that the bladder volume at diagnosis was the best single predictor of a severely decreased estimated glomerular filtration rate in LUTO fetuses referred before the 26th week of gestation. Moreover, an increased bladder volume was also correlated with oligohydramnios in our cohort, and oligohydramnios was reported to predict postnatal renal impairment with a positive likelihood ratio of 17.0 [27] Such impairment of renal function can result in cardiac dysfunction via the abovementioned pathways.

The potential limitation of this study was the absence of invasive fetal urine/serum biometry due to the lack of fetal intervention in our region. However, we measured a correlation between ultrasound features and cardiac deformation, which could provide more useful information for prenatal counseling for most developing countries and regions. In addition, it is necessary to further examine the characteristics of myocardial deformation in LUTO along the duration of pregnancy as well as postinterventionally.

## Conclusions

In summary, the strain and strain rate of both ventricles were decreased in fetuses with isolated LUTO, and such cardiac dysfunction was correlated with the urinary bladder volume. Evaluating the myocardial deformation in fetal LUTO could provide information to help in parental counselling and intervention monitoring.

## Data Availability

The datasets used and/or analysed during the current study are available from the corresponding author on reasonable request.

## Abbreviations

LUTO: Lower urinary tract obstruction
S: Strain
SRs: Strain rate for systole
SRd: Strain rate for diastole
